# Downregulation of NEDD4L by EGFR signaling promotes the development of lung adenocarcinoma

**DOI:** 10.1186/s12967-022-03247-4

**Published:** 2022-01-28

**Authors:** Guoyin Li, Zewen Song, Changjing Wu, XiaoYan Li, Liping Zhao, Binghua Tong, Zhenni Guo, Meiqing Sun, Jin Zhao, Huina Zhang, Lintao Jia, Shengqing Li, Lei Wang

**Affiliations:** 1 College of Life Science and Agronomy, Zhoukou Normal University, Zhoukou, China; 2 State Key Laboratory of Cancer Biology, Department of Biochemistry and Molecular Biology, Fourth Military Medical University, 169 Changle West Road, Xi’an, 710032 China; 3 Academy of Medical Science, Zhengzhou University, Zhengzhou, China; 4 Department of Oncology, The Third Xiangya Hospital of Central South University, Changsha, China; 5 Department of Blood Transfusion, Shanxi Province People’s Hospital, Taiyuan, China; 6 Department of Pathology, Shanxi Province People’s Hospital, Taiyuan, China; 7 Department of Pulmonary and Critical Care Medicine, Huashan Hospital, Fudan University, Shanghai, 200040 China; 8 Clinical Medical College, Yangzhou University, Yangzhou, China

**Keywords:** NEDD4L, Lung adenocarcinoma, mTOR, EGFR, Development

## Abstract

Cumulative evidence indicates that the abnormal regulation of the NEDD4 family of E3-ubiquitin ligases participates in the tumorigenesis and development of cancer. However, their role in lung adenocarcinoma (LUAD) remains unclear. This study comprehensively analyzed the NEDD4 family in LUAD data sets from public databases and found only NEDD4L was associated with the overall survival of LUAD patients. Gene set enrichment analysis (GSEA) indicated that NEDD4L might be involved in the regulation of mTORC1 pathway. Both cytological and clinical assays showed that NEDD4L inhibited the activity of the mTOR signaling pathway. In vivo and in vitro experiments showed that NEDD4L could significantly inhibit the proliferation of LUAD cells. In addition, this study also found that the expression of NEDD4L was regulated by EGFR signaling. These findings firstly revealed that NEDD4L mediates an interplay between EGFR and mTOR pathways in LUAD, and suggest that NEDD4L held great potential as a novel biomarker and therapeutic target for LUAD.

**Supplementary Information:**

The online version contains supplementary material available at 10.1186/s12967-022-03247-4.

## Introduction

The morbidity and mortality of lung cancer rank first among all the malignant tumors worldwide [1]. Although significant progress has been made in tumor screening and treatment in recent years, the 5-year survival rate for lung cancer has not improved significantly [2]. Lung adenocarcinoma (LUAD), accounting for 50% of all lung cancer cases, is the most common subtype of non-small cell lung cancer (NSCLC) [3]. Therefore, an in-depth study of the pathogenesis of LUAD is essential for the development of its biomarkers and the improvement of the detection and survival rate of LUAD patients.

NEDD4 family belongs to the E3 ubiquitin ligase subfamily, which contains nine members: NEDD4, NEDD4L, WWP1, WWP2, HECW1, HECW2, SMURF1, SMURF2, and ITCH. NEDD4-like proteins can target cell cycle and apoptosis-related proteins and regulate various biological processes of cells [4]. Previous studies have shown that NEDD4-like proteins can regulate TGFβ, Notch, Hedgehog, Hippo, Wnt, Raf/ MEK/ERK, and PTEN/PI3K/Akt signaling pathways, which play key roles in the occurrence and development of carcinomas [5]. NEDD4 was confirmed to mediate cell migration signaling of EGFR in lung cancer [6]. NEDD4L was reported to inhibit TGFβ induced epithelial to mesenchymal transition (EMT) and be related to the prognosis of lung cancer patients [7, 8]. WWP1 induces the ubiquitination of EGFR at Lys689, which enhances EGFR recycling in lung cancer [9]. WWP2 promotes the proliferation of lung cancer cells by mediating p27 ubiquitination [10]. SMURF1 regulates lung cancer proliferation and migration by mediating PIPKIγ ubiquitination [11]. SMURF2 acts as a negative regulator of TGFβ signaling by ubiquitin-mediated degradation of TGFβR1 in lung cancer [12]. HECW1 promotes the metastasis of NSCLC by mediating the ubiquitination of Smad4 [13]. Li et al. reported that inhibition of ITCH could suppress proliferation and induce apoptosis of lung cancer cells [14]. The function of HECW2 in lung cancer is still unclear. NEDD4 family members play an important role in the biological process of lung cancer, but their function and mechanism in LUAD still need to be further explored.

EGFR is the key driver of lung cancer. EGFR signaling network plays a vital role in the maintenance and growth of epithelial tissues [15]. EGFR signaling pathway is involved in the regulation of lung cancer cell proliferation, migration, invasion, apoptosis, and other biological processes [16–18]. mTOR is another key oncogene in carcinomas. Activated mTOR signaling regulates protein synthesis, survival, and cell growth by downstream effectors P70S6K, 4ebp1, and eIF4 [19]. Insulin inhibits TSC2 by activating Akt and releases the inhibitory effect of the latter on Rheb, thereby activating mTOR [20]. In addition, PI3K Signaling also regulates mTORC1 through Rheb [21]. However, the activity of the PI3K/Akt pathway is regulated by EGFR. Due to the limited role of TSC2 in lung adenocarcinoma, EGFR should be able to regulate mTOR activity in other ways. Deng et al. reported that ubiquitin ligases RNF152 and USP4 play a vital role in Rheb-mediate mTORC1 activation [22]. Hong's study showed that CBLC enhanced EGFR dysregulation and signaling in LUAD [23].

Cumulative evidence indicates that ubiquitin ligase is related to the EGFR signal and the mTOR pathway. However, the role of the NEDD4 family in LUAD remains unclear and needs to be further studied.

In this study, we probed the expression of NEDD4 family members in LUAD via bioinformatics analyses and found that NEDD4L was down-regulated and associated with poor prognosis. Subsequently, we confirmed that EGFR enhanced mTOR signaling activity by down-regulating NEDD4L, thereby promoting the proliferation of LUAD.

This study is first to reveal the mechanism of EGFR/NEDD4L/mTOR signal axis, and provided a new potential target for the treatment of LUAD.

## Materials and methods

### Data acquisition and processing

The LUAD project of the TCGA (TCGA_LUAD) dataset and GSE68465 dataset was acquired from the GDC hub of the UCSC Xena website (http://xena.ucsc.edu/public) and the Gene Expression Omnibus (GEO) database (https://www.ncbi.nlm.nih.gov/gds/), respectively. Data were processed as described in our previous study [24].

### Online database analysis

A meta-analysis of NEDD4L transcriptional level in LUAD was performed by the Oncomine database (https://www.oncomine.org/resource/login.html). NEDD4L expression data of 9 groups of LUAD and normal tissues were retrieved and compared statistically. The default threshold was as follows: P < 1E−4, fold change > 2, and the top 10% of genes. The GEPIA database (http://gepia.cancer-pku.cn/index.html) was used to analyze the correlation between NEDD4L expression level and prognosis of LUAD patients.

### Cell culture and treatment

Human LUAD cancer H1299 and PC9 cells were obtained from Cell Bank of Shanghai Institute for Biological Sciences, Chinese Academy of Sciences. Cells were cultured in RPMI 1640 (H1299), DMEM (PC9) medium, containing 10% FBS, and maintained in an incubator with constant temperature and CO_2_. Cells were treated with gefitinib (ZD1839, Selleck).

### Transfection and lentiviral transduction

Transfection was performed using Lipo8000™ (C0533, Beyotime). The siRNAs were acquired from GenePharma Company (Shanghai, China). The siRNA sequences are as follows: siEGFR#1, 5′-GUCCGCAAGUGUAAGAAGUTT-3′; siEGFR#2, 5′-GGAGAUAAGUGAUGGAGAUTT-3′; negative control, 5′-UUCUCCGAACGUGUCACGUTT-3′. The lentivirus was acquired from Genechem company (Shanghai, China).

The target sequence of shNEDD4L is as follow: 5′-GGAACUAAGCAGAAGGCUUTT-3′. The method of lentivirus transfection of cells was described in our previous study [25].

### CCK-8 assay

The cells were placed in 96 well plates and detected at 24, 48, and 72 h by Cell Counting Kit-8 (Beyotime C0038, China) [26]. The cell culture medium and CCK-8 reagent were prepared into working solution according to 10:1. Remove the residual medium and add 100 μL working solution, incubate at 37℃ for 1 h, and detect with enzyme labeling instrument (Biorad 680, USA).

### Colony formation assay

The PC9 cells were seeded onto 6-well plates (200 cells/well) and cultured for 14 days. Cells were fixed using paraformaldehyde and stained using crystal violet as described in our previous study [26]. Each group had three repetitions, and the t-test was used to analyze the difference in clone number between groups.

### Cell cycle assay

Cells were first starved in 1% FBS medium for 12 h, then spread onto 6-well plates and cultured in complete medium for 24 h. Cells were treated using a cell cycle and apoptosis analysis kit (C1052, Beyotime) and detected using flow cytometry. The difference between groups was detected by t-test.

### Gene set enrichment analysis

Patients from the TCGA_LUAD dataset were divided into high-expression and low-expression subgroups according to NEDD4L expression level. Gene set enrichment analysis (GSEA) was performed to investigate pathways enriched in the high-expression and low-expression subgroups. C2.cp.k*egg.v7.1.symbols.gmt* was chosen as the gene set database. Signaling pathways that meet the following criteria can be significantly enriched: nominal p-value < 0.05, q-value < 0.25, and normalized enrichment score > 1 [27].

### Western blotting

Cells were lysed on ice with pre-cooled RIPA for 10 min, and centrifuged at 4 °C for 10 min (12,000 rpm/min) to obtain the supernatant for subsequent detection. Western blotting was performed using antibodies against EGFR (#4267S, CST), phospho-EGFR (#3777, CST), mTOR (#2983, CST), phospho-mTOR (ab109268, Abcam), NEDD4L (ab46521, Abcam), and S6K (ab186753, Abcam), phospho-S6K (ab131436, Abcam), β-Actin (#3700, CST). Horseradish peroxidase-labeled Goat anti-rabbit IgG (H + L) (A0208, Beyotime) was used as secondary antibodies.

### Patients and specimens

Twenty-four pairs of human LUAD tissues and para-carcinoma tissues were obtained from patients at Shanxi Provincial People's Hospital (Taiyuan, China). The use of clinical specimens was approved by the Ethics Committee of Zhoukou Normal University (ZKNU-2019043).

### Immunohistochemistry and immunofluorescence assay

The carcinoma tissues and para-carcinoma tissues were fabricated into a tissue chip. Immunohistochemical (IHC) staining of FFPE sections was performed as described [26]. The expression of target genes was assessed by the H-score system, and the formula for the H-score is as follows: Histoscore = Σ (I × Pi), where I = intensity of staining and Pi = percentage of stained tumor cells [28]. Cells were seeded onto the special dish for laser confocal scanning, and the density was about 30%. Cells were treated with 10 μM gefitinib for 24 h. Immunofluorescence was performed as described in our previous study [26].

### Xenograft lung adenocarcinoma model

For the xenograft lung cancer models, 6-week-old BALB/c nude mice were obtained from Weitong Lihua Experimental Animal Technology Co., Ltd (Beijing, China). H1299 cells (5 × 10^6^ per mouse) were injected subcutaneously into the right side of mice, and the tumor volumes were measured 7 days later (Day 0). We measured the tumor volume every three days, and the formula is as follows: volume = (length*width^2^)/2. On day 18 of tumor development, mice were sacrificed and tumors were isolated and subjected to IHC analysis. All animal experiments were approved by the Ethics Committee of Zhoukou Normal University (ZKNU-2019043).

### Statistical analysis

Correlation analysis was conducted by GraphPad Prism (version 8.0.1) with the Pearson method. The IHC score difference analysis was also conducted by GraphPad Prism software using paired t-test. The prognosis of LUAD patients from different subgroups was compared using the Kaplan–Meier method with a log-rank test. Target genes expression from different groups was compared by R software (version 4.0.2) using the Wilcox test. The “pheatmap”, "ggplot2", "ggpubr", "survival", "survminer", "plyr", "grid", and "gridExtra" packages in R (version 4.0.2) were used for visualization. A p-value of less than 0.05 was considered to be statistically significant (*, p < 0.05; **, p < 0.01; ***, p < 0.001).

## Results

### NEDD4L downregulation is correlated with poor prognosis in LUAD

**Fig. 1:**
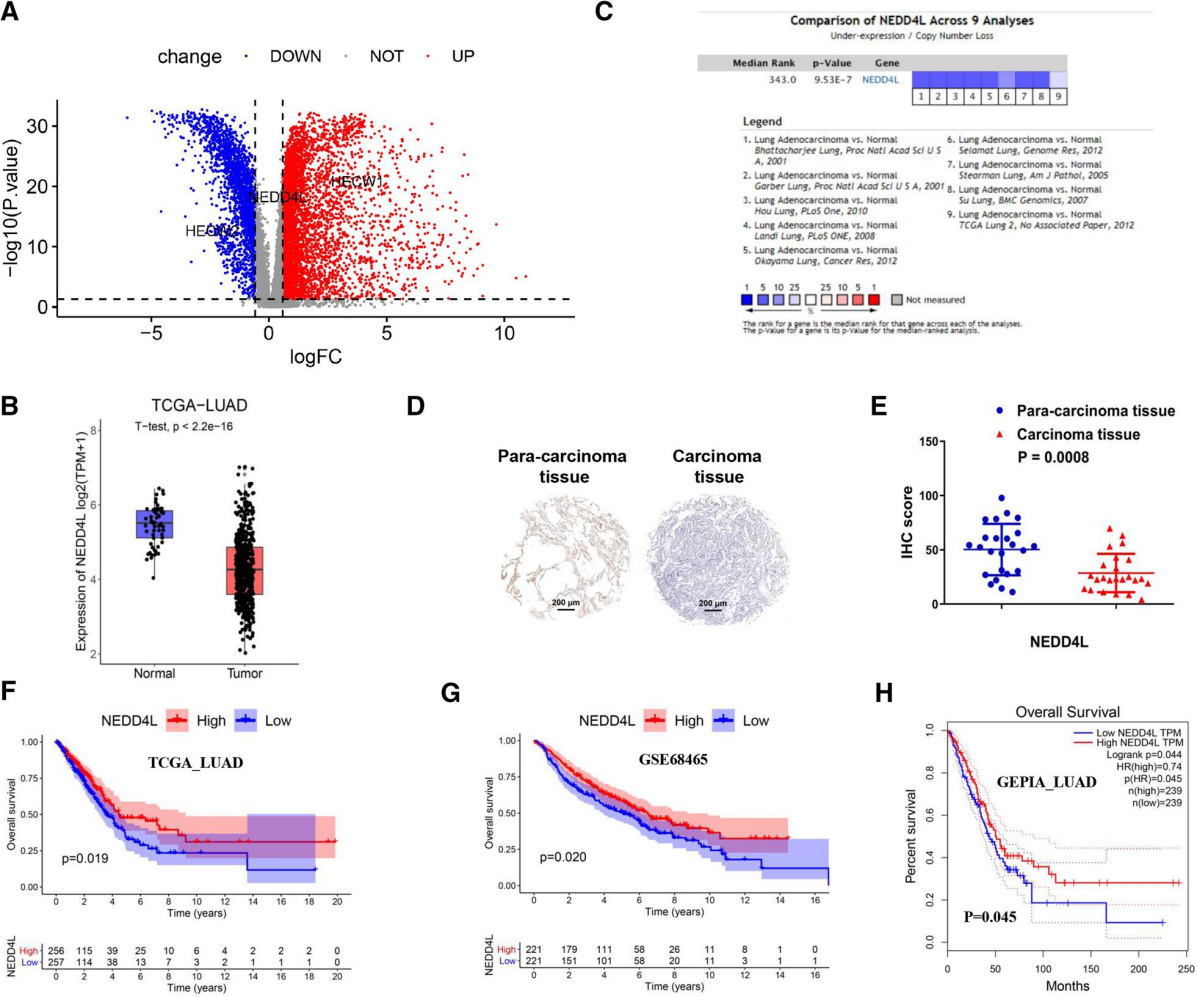
Expression and prognostic analysis of NEDD4L in LUAD. **A** The volcano plot for differentially expressed genes (DEGs) (FC > 1.5 and adjusted p-value < 0.05). **B** Relative mRNA level of NEDD4L in normal and tumor tissues from the TCGA_LUAD dataset. **C** Meta-analysis of NEDD4L using the Oncomine database showed that NEDD4L was significantly downregulated in LUAD tissues. **D** Representative IHC staining of NEDD4L in carcinoma and para-carcinoma tissues. **E** Relative protein levels of NEDD4L in carcinoma and para-carcinoma tissues. **F**–**H** Kaplan–Meier curves of the OS of the patients from the TCGA_LUAD, GSE68465, and GEPIA_LUAD database

To investigate the potential function of the NEDD4 family members in LUAD, we examined their transcription levels in 59 normal tissues and 526 cancer tissues from the TCGA_LUAD dataset. The differentially expressed genes (DEGs) between normal and tumor tissues were analyzed using the following criteria: fold change (FC) > 1.5 and p < 0.05. The results revealed that NEDD4L and HECW2 were significantly downregulated while HECW1 was significantly upregulated in cancer tissues compared with normal tissues (Fig. 1A, B). Subsequently, a meta-analysis of NEDD4L transcriptional level in LUAD was performed using 9 studies from the Oncomine database, and the result was consistent with that of the TCGA_LUAD dataset (Fig. 1C). Next, we examined NEDD4L protein levels in LUAD tissues and para-carcinoma tissues using IHC. The result confirmed that NEDD4L was significantly downregulated in LUAD (Fig. 1D, E). Survival analysis of patients from the TCGA_LUAD dataset indicated that only NEDD4L was associated with overall survival (OS) of patients, and NEDD4L patients expressing higher levels of NEDD4L showed better prognosis (Fig. 1F, Additional file 1: Fig. S1). Analyses using other datasets (GSE48465 and GEPIA_LUAD) validated that NEDD4L could serve as a better prognosis of LUAD (Fig. 1G, H). Taken together, these findings suggested that NEDD4L might function as a tumor suppressor in clinical LUAD.

### NEDD4L represses the proliferation of LUAD cells

**Fig. 2:**
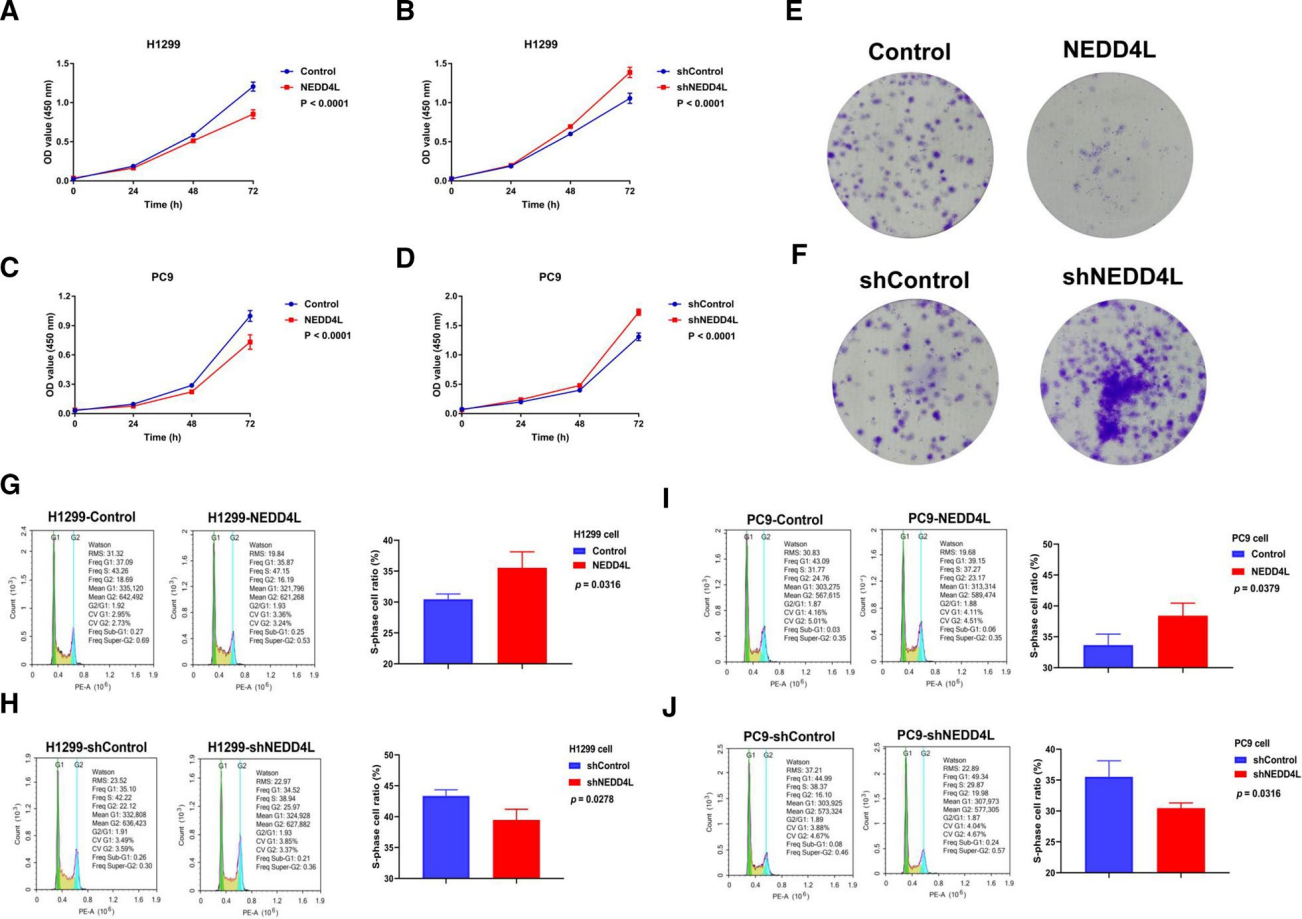
NEDD4L downregulation contributed to LUAD cell proliferation. **A**–**D** CCK-8 assays for LUAD cells stably overexpressing NEDD4L (**A**, **C**) or stably knocking down NEDD4L (**B**, **D**). **E**, **F** Colony formation assays for PC9 cells, which were stably overexpressing NEDD4L (**E**) or stably knocking down NEDD4L (**F**). **G**–**J** LUAD cells stably overexpressing NEDD4L (**G**, **I**) or stably knocking down NEDD4L (**H**, **J**) subjected to flow cytometry analysis for detecting cell cycle

To explore the function of NEDD4L in lung adenocarcinoma, we carried out the following cytological experiments. CCK-8 and clone formation assays were performed to detect cells proliferation ability. Overexpression of NEDD4L in LUAD cell lines inhibited cells proliferation, while knockdown of NEDD4L promoted cell proliferation (Fig. 2A–D). NEDD4L knockdown promoted in vitro colony formation of PC9 cells, while NEDD4L overexpression inhibited colony formation (Fig. 2E, F). Flow cytometry results showed that the number of S-phase cells increased after NEDD4L overexpression, while it decreased after NEDD4L knockdown (Fig. 2G–J). These results suggested that NEDD4L might inhibit the proliferation of LUAD cells by inducing cell cycle arrest in S phase.

### NEDD4L inhibited mTOR pathway activity in LUAD

**Fig. 3:**
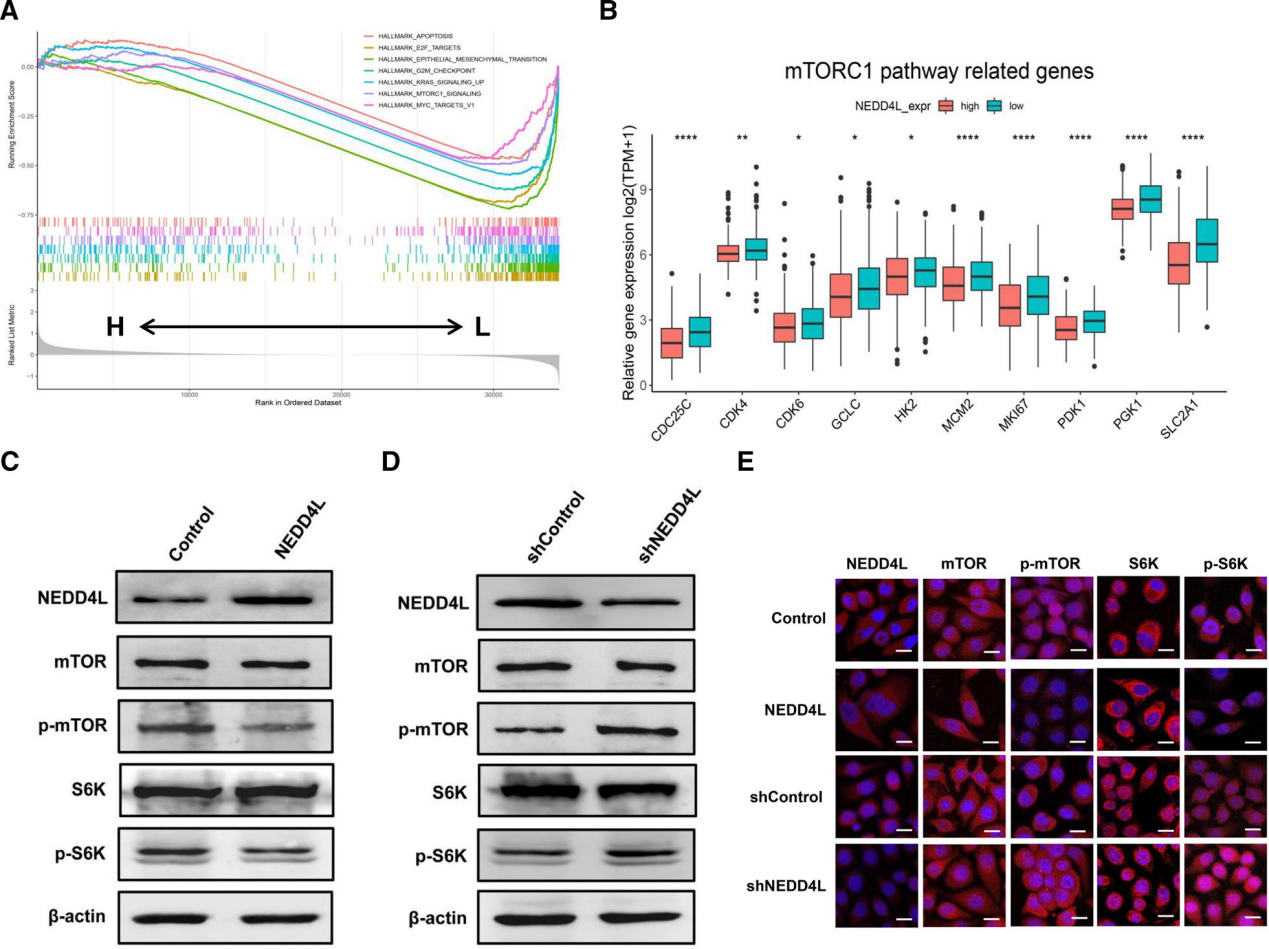
NEDD4L regulated the activity of the mTOR pathway in LUAD cells. **A** GSEA results indicated that genes in the NEDD4L low expression subgroup were enriched in the mTORC1 pathway. **B** Relative mRNA levels of mTORC1 pathway-related genes in samples from the TCGA_LUAD dataset. (C&D) Western blot analysis of PC9 cells stably overexpressing NEDD4L (**C**) or stably knocking down NEDD4L (**D**). **E** Immunofluorescence microscopy analysis of PC9 cells stably overexpressing NEDD4L or stably knocking down NEDD4L

**Fig. 4:**
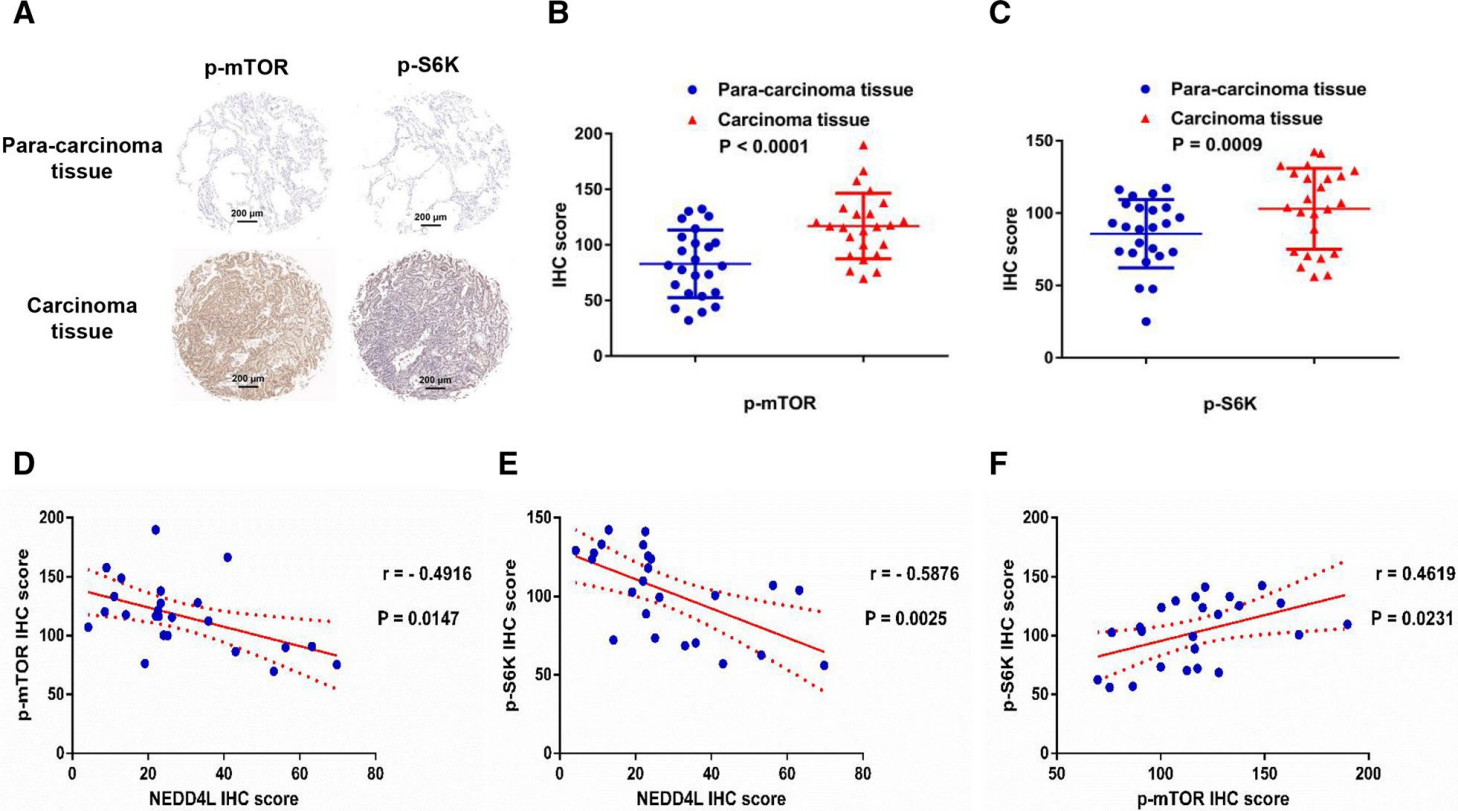
NEDD4L was negatively correlated with p-mTOR and p-S6K in LUAD tissues. **A** Representative IHC staining of p-mTOR and p-S6K in carcinoma and para-carcinoma tissues. **B**, **C** Relative protein levels of p-mTOR (**B**) and p-S6K (**C**) in carcinoma and para-carcinoma tissues. **D**–**F** Correlation analysis of NEDD4L, p-mTOR, and p-S6K

Because the mTOR signaling pathway is closely related to tumor proliferation [29], we explored the effect of NEDD4L on its activity in LUAD cells. GSEA results showed that mTORC1 signaling was einforced in the NEDD4L low-expression subgroup (Fig. 3A). Compared with the NEDD4L high-expression subgroup, the mTORC1 pathway-related genes such as *CDC25C*, *CDK4*, *CDK6*, *GCLC*, *HK2*, *MCM2*, *MKI67*, *PDK1*, *PGK1*, and *SLC2A1* were upregulated in the NEDD4L low-expression subgroup (Fig. 3B). Western blotting (WB) and cellular immunofluorescence (ICC) results showed that NEDD4L overexpression reduced the levels of phosphorylated mTOR (p-mTOR) and S6K (p-S6K), while NEDD4L knockdown promoted the phosphorylation of these proteins (Fig. 3C–E). Immunohistochemistry (IHC) results indicated that p-mTOR and p-S6K were significantly upregulated in carcinoma tissues, and significantly negatively correlated to NEDD4L (Fig. 4A–F). These data suggest that the mTOR pathway could be regulated by NEDD4L in LUAD.

### NEDD4L expression was deregulated by EGFR in LUAD

**Fig. 5:**
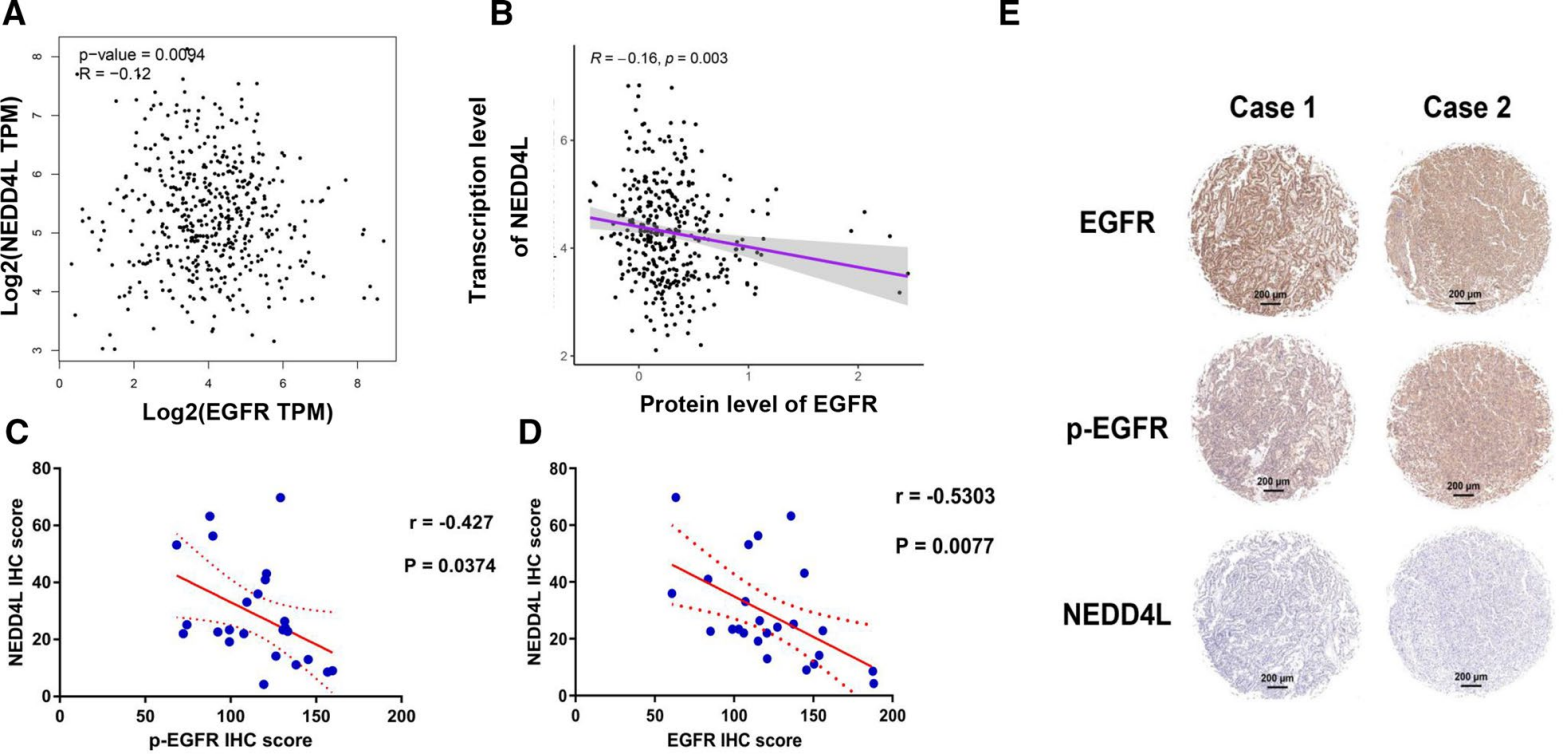
EGFR was negatively correlated with NEDD4L in LUAD tissues. Bioinformatics analysis of the TCGA_LUAD dataset showed that NEDD4L was negatively correlated with EGFR at the mRNA level (**A**) and protein level (**B**). IHC score showed that NEDD4L was negatively correlated with EGFR (**C**) and p-EGFR (**D**). **E** Representative IHC staining of indicated proteins in carcinoma and para-carcinoma tissues

**Fig. 6:**
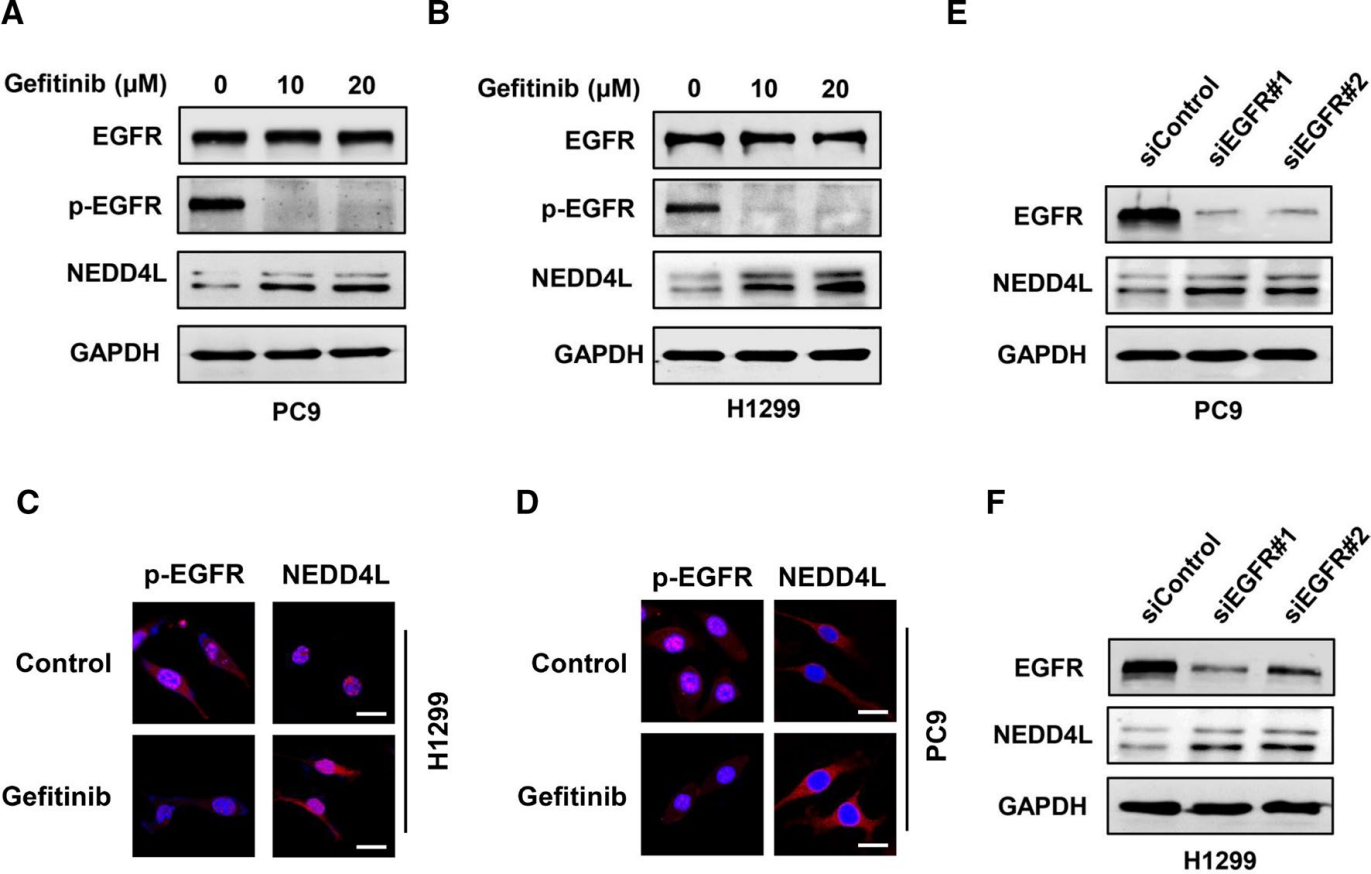
EGFR signaling suppressed the expression of NEDD4L in LUAD cells. **A**, **B** LUAD cells were treated with the indicated dose of gefitinib and subject to Western blot analysis. **C**, **D** LUAD cells were treated with 10 μM of gefitinib and subject to immunofluorescence microscopy analysis. **E**, **F** LUAD cells were transfected with scrambled or EGFR-target siRNAs and subject to Western blot analysis

Since EGFR is the key driver of lung cancer and can regulate the expression of various genes, therefore we analyzed the correlation between EGFR and NEDD4L. As a result, NEDD4L was negatively correlated to EGFR mRNA and protein levels in cancer tissues in the TCGA_LUAD dataset (Fig. 5A, B). However, there was a significant positive correlation between NEDD4L and EGFR in normal tissues (Additional file 2: Fig. S2). IHC was then performed to detect the expression of EGFR, p-EGFR, and NEDD4L in 24 LUAD samples. Correlation analysis showed that NEDD4L was significantly negatively correlated with total and phosphorylated EGFR (Fig. 5C, D). To probe whether NEDD4L expression was regulated by the EGFR pathway, we treated LUAD cells with gefitinib, a tyrosine kinase inhibitor targeting EGFR. WB and ICC results showed that gefitinib could significantly inhibit the activity of EGFR and promote NEDD4L expression (Fig. 6A–D). We also knocked down EGFR in LUAD cells by siRNA, and WB assay indicated that the expression level of NEDD4L was upregulated after EGFR knockdown (Fig. 6E, F). These results suggest that the EGFR pathway could inhibit NEDD4L expression in LUAD.

### NEDD4L inhibited the proliferation of LUAD in vivo

**Fig. 7:**
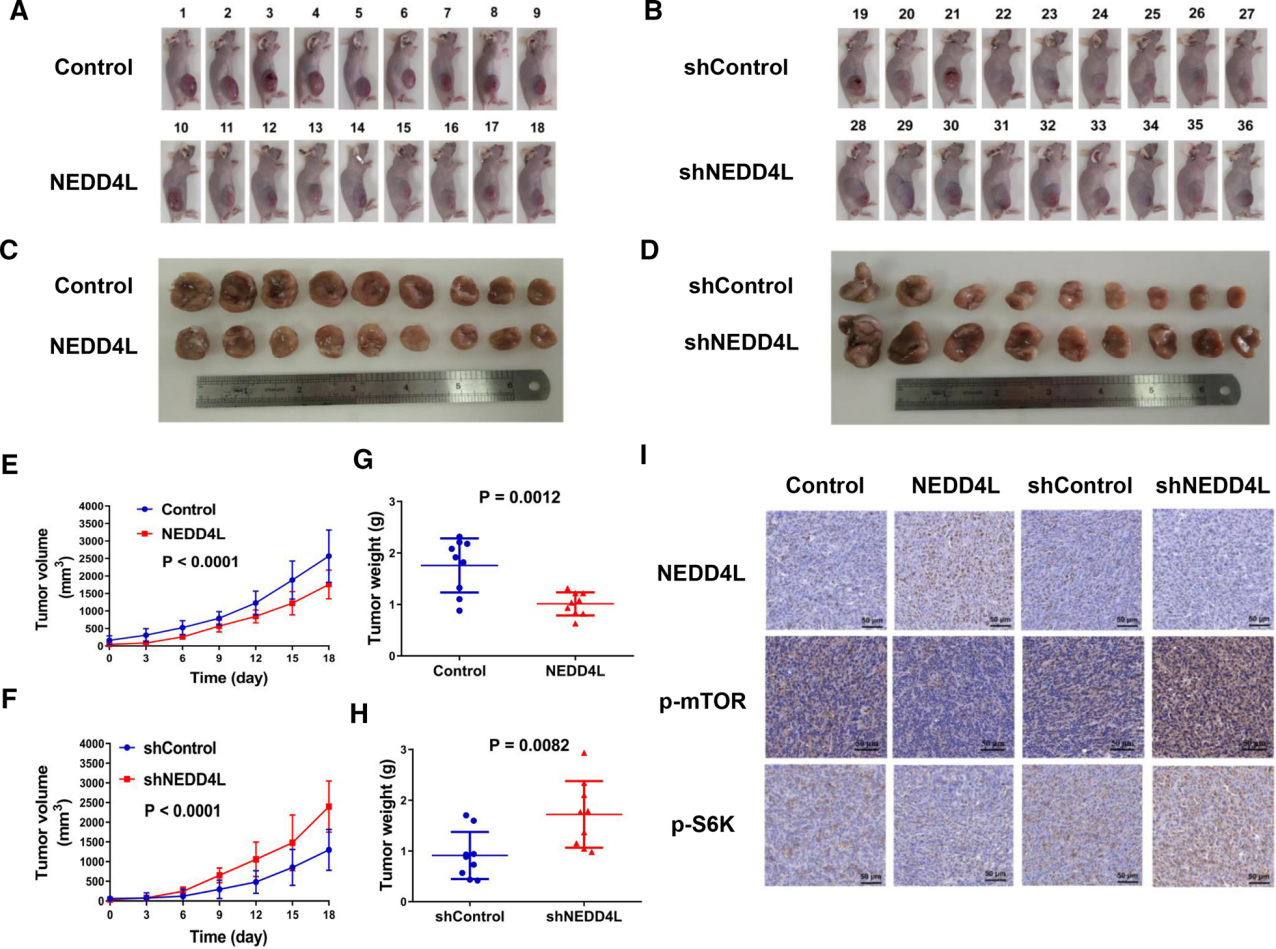
NEDD4L suppressed the proliferation of breast cancer in vivo. **A**, **B** Xenograft models of LUAD cancers derived from H1299-shControl or H1299-shNEDD4L or H1299-Control or H1299-NEDD4L cells were established as described in “Materials and methods” section. **C**, **D** Tumors were obtained after mice were sacrificed on Day 18. (E&F) The tumor volume was measured every three days and the growth curves were drawn. **G**, **H** Scatter plots of tumor weight on Day 18. **I** Immunohistochemical results of tumor tissue from the xenograft models

To further the role of NEDD4L in mTOR signaling and LUAD pathogenesis, we generated a xenograft LUAD model through subcutaneous injection of nude mice with H1299 cells (Fig. 7A, B). We measured the tumor volume every three days until the mice died, and plotted the tumor growth curve. The results showed that NEDD4L overexpression significantly inhibited tumor growth in vivo, while knockdown of NEDD4L remarkably promoted tumor proliferation (Fig. 7A–G). We harvested the tumors after the mice were sacrificed and weighted them. Comparing with control groups, we found that the weight of the NEDD4L overexpression group was significantly decreased, while that of the NEDD4L knockdown group was significantly increased (Fig. 7G, H). The tumor tissues were cut into paraffin sections, and subject to IHC analyses. The results showed that the levels of p-mTOR and p-S6K were significantly reduced in NEDD4L overexpression samples, while they were significantly upregulated in NEDD4L knockdown samples (Fig. 7I). Thus, these data suggest that the EGFR-NEDD4L-mTOR axis is crucial for the development of LUAD.

## Discussion

NEDD4 family ubiquitin ligases, as the key regulators of cell surface receptor signaling, play a key role in the development and progression of tumors [30, 31]. In addition, NEDD4-like proteins have high substrate specificity, which makes them potentially suitable targets for tumor therapy [32]. However, the detailed role of NEDD4-like proteins in LUAD pathogenesis is still unclear. Here, our integrated bioinformatics analysis demonstrated a prognostic value of NEDD4L in LUAD, which was consistent with the results observed by Yang et al. in lung cancer [8]. GSEA results indicated that genes regulated by NEDD4L were enriched in the mTORC1 pathway (Fig. 3A, B). Subsequently, we knocked down or overexpressed NEDD4L in LUAD cells and measured the activity of the mTOR pathway. The results suggested that NEDD4L played a critical role in the regulation of the mTOR pathway, which was consistent with Chelly's study [33]. To confirm the reliability of the conclusion, IHC was performed using human LUAD tissues and mouse xenograft tumor tissues, and which showed that NEDD4L expression level was significantly negatively correlated with p-mTOR and p-S6K (Figs. 4D, E, 7I, J). The mTOR pathway plays a decisive role in cell proliferation, survival, and metabolism, which suggests that NEDD4L may be related to the development of LUAD [34]. Wang et al. showed that NEDD4L suppressed proliferation and migration of NSCLC [35]. Our results of in vitro and in in vivo experiments indicated that NEDD4L inhibited the proliferation of LUAD by inducing S phase arrest (Figs. 2, 7A–H). Together, these results suggest that NEDD4L acted as a tumor suppressor gene in LUAD.

EGFR signaling is associated with the malignant behaviors of lung cancer and drives uncontrolled cell growth and invasion [36]. However, it remains to be fully characterized how EGFR signaling regulates target genes and drives the development of LUAD. We found that the levels of total and phosphoryltaed EGFR were negatively correlated with the expression of NEDD4L in LUAD tissues (Fig. 5). The results of our in vitro experiments indicated that EGFR inhibited the expression of NEDD4L in LUAD cells (Fig. 6). However, bioinformatics analysis showed that EGFR was positively correlated to NEDD4L in normal tissues (Additional file 2: Fig. S2). The abnormal activation of EGFR signaling in LUAD may lead to changes in the expression or activity of a cohort of transcription factors like SP1 [37], AP-1 [38] and CREB [39], all of which were documented to that regulate NEDD4L expression. Further investigations are needed to define the mechanism underlying transcriptional regulation of NEDD4L by EGFR in LUAD cells.

Understanding the complicated cross-talk between different signaling pathways during tumorigenesis will contribute to the development of new methods and new targets for tumor therapy. The mTOR and EGFR signaling pathways are crucial for LUAD. However, whether they synergistically promote carcinogenesis or whether there is an interplay between these canonical pathways in lung cancer is still unclear. Kumar et al*.* showed that the mTOR‑Rictor‑EGFR axis played a vital role in glioblastoma [40]. Cui et al*.* reported that EGFR promoted tumor proliferation and angiogenesis by activating the PI3K-AKT-mTOR and RAF-MEK-ERK pathways in hepatocellular carcinoma [41]. Interestingly, in this study, we found that NEDD4L could act as a bridge between EGFR and mTOR pathways. Combining the existing literature and our findings, we propose that the EGFR-NEDD4L-mTOR axis plays a vital role in thedevelopment of LUAD, suggesting a prognostic role of NEDD4L in EGFR-driven lung malignancy as well as the applicability of combined EGFR and mTOR targeting in LUAD therapy.

## Supplementary Information

**Additional file 1: Figure S1.** Correlation between NEDD4 family and OS of LUAD patients. (A-H) Kaplan–Meier curves of the OS of the patients from the TCGA_LUAD database.

**Additional file 2: Figure S2.** NEDD4L was significantly positively correlated with EGFR in normal tissues.

## Data Availability

The datasets generated during and/or analyzed during the present study are available from the corresponding author on reasonable request.
