# Supplementary figures and images for: Downregulation of NEDD4L by EGFR signaling promotes the development of lung adenocarcinoma
Source: J Transl Med. 2022 Jan 28;20:47. doi: 10.1186/s12967-022-03247-4 (PMC8800232; doi:10.1186/s12967-022-03247-4)

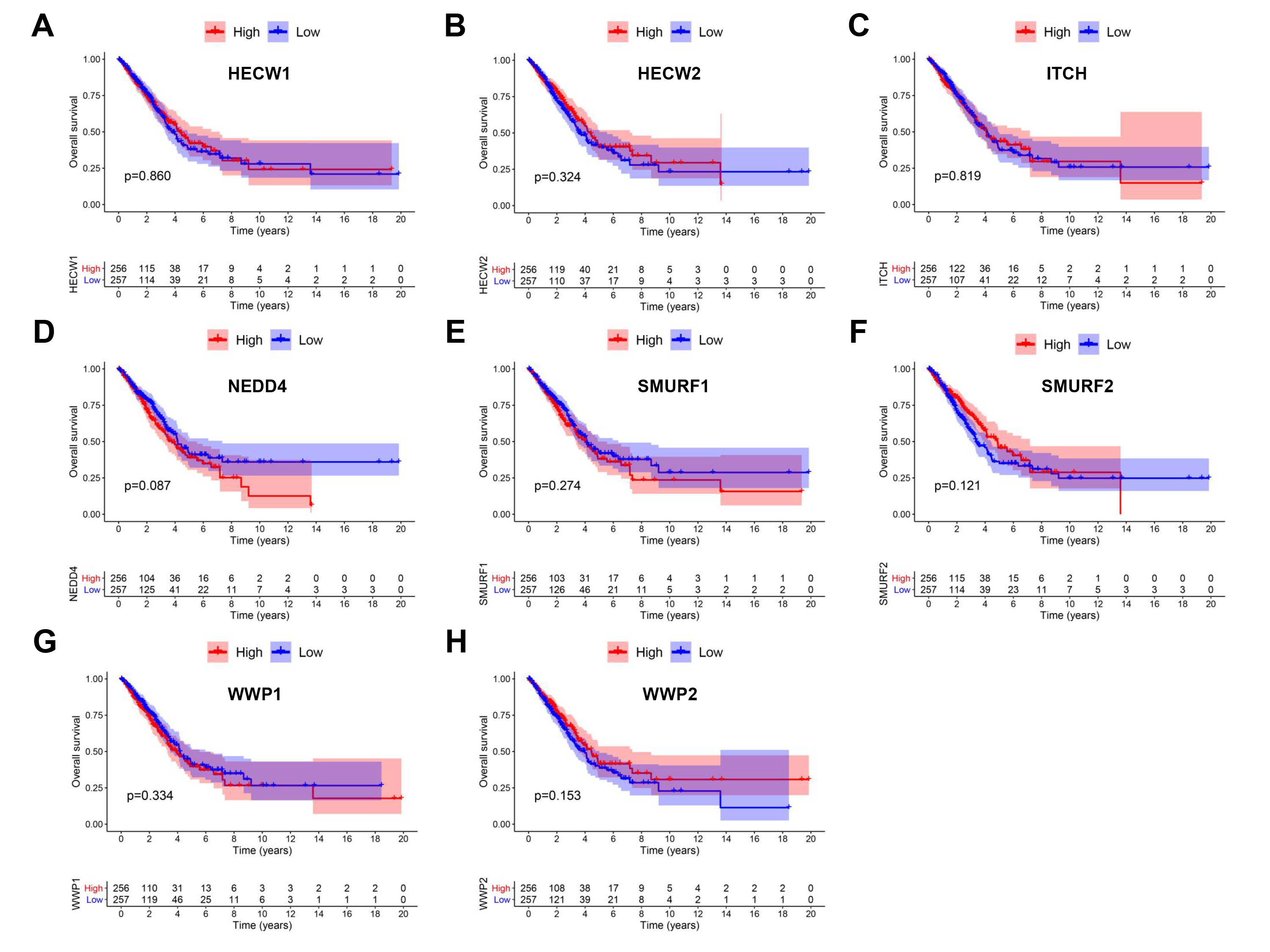

Supplement: Supplementary file 1 — Additional file 1: Figure S1. Correlation between NEDD4 family and OS of LUAD patients. (A-H) Kaplan–Meier curves of the OS of the patients from the TCGA_LUAD database. [file 12967_2022_3247_MOESM1_ESM.png]

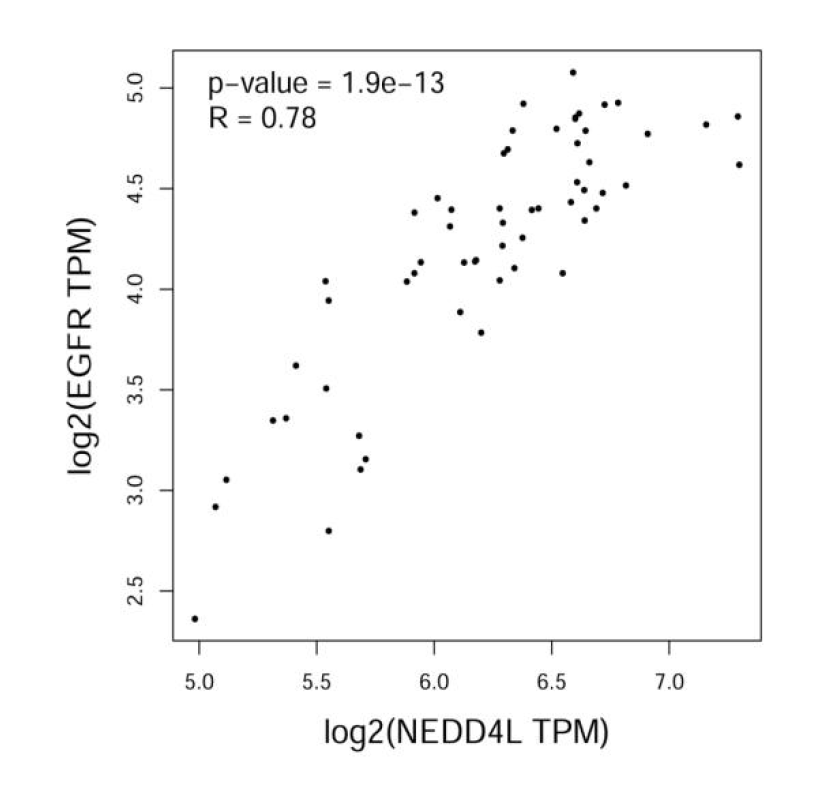

Supplement: Supplementary file 2 — Additional file 2: Figure S2. NEDD4L was significantly positively correlated with EGFR in normal tissues. [file 12967_2022_3247_MOESM2_ESM.png]
